# Multi-Camera and Structured-Light Vision System (MSVS) for Dynamic High-Accuracy 3D Measurements of Railway Tunnels

**DOI:** 10.3390/s150408664

**Published:** 2015-04-14

**Authors:** Dong Zhan, Long Yu, Jian Xiao, Tanglong Chen

**Affiliations:** School of Electrical Engineering, Southwest Jiaotong University, Erhuan Road No. 111, Jinniu District, Chengdu 610031, China; E-Mails: yulong.swjtu@163.com (L.Y.); jxiao@swjtu.edu.cn (J.X.); tl_chen@126.com (T.C.)

**Keywords:** railway tunnel, 3D clearance, dynamic inspection

## Abstract

Railway tunnel 3D clearance inspection is critical to guaranteeing railway operation safety. However, it is a challenge to inspect railway tunnel 3D clearance using a vision system, because both the spatial range and field of view (FOV) of such measurements are quite large. This paper summarizes our work on dynamic railway tunnel 3D clearance inspection based on a multi-camera and structured-light vision system (MSVS). First, the configuration of the MSVS is described. Then, the global calibration for the MSVS is discussed in detail. The onboard vision system is mounted on a dedicated vehicle and is expected to suffer from multiple degrees of freedom vibrations brought about by the running vehicle. Any small vibration can result in substantial measurement errors. In order to overcome this problem, a vehicle motion deviation rectifying method is investigated. Experiments using the vision inspection system are conducted with satisfactory online measurement results.

## 1. Introduction

With the speed of trains increasing and the departure frequency improving, railway infrastructure quality requires a more proper and effective maintenance system to guarantee its operational security. Railway tunnels, as an important part of railway infrastructure, have critical dimension limitations regarding their 3D clearance. The physical dimensions of a railway tunnel must be in strict accordance with relevant standards [1]. In order to determine the effective clearance of operating trains and to ensure the safety of the railway system, regular monitoring of railway tunnel 3D clearance needs to be implemented. A slight deformation found just at an early stage allows for better scheduling of the maintenance, which can eliminate potential risks, avoid incidents and also reduce maintenance costs.

The existing techniques for railway tunnel 3D clearance inspection can roughly be divided into contact and non-contact measurements. In the former category, the 3D railway tunnel clearance metrics information is acquired by skilled workers utilizing a special mechanical gauge. This has the advantages of low-cost and simple implementation; however, this approach is extremely laborious and time consuming, and it can only satisfy the local static measurement demands and can hardly estimate any potential trends of railway tunnel deformation with time.

With the development of computer vision and image processing techniques, methods of on-line dynamic inspection for railway tunnel 3D clearance have appeared with the help of non-contact vision sensors mounted on dedicated vehicles. Methods of non-contact dynamic measurements for railway tunnel 3D clearance commonly in use include time of flight (TOF) inspection, stereoscopic vision (SV) inspection and laser triangulation (LT) inspection [2,3,4,5]. The TOF method refers to the time it takes for a pulse of energy to travel from its transmitter to the surface of an object and then back to the receiver. The emitted light is used as the energy source, and the relevant parameter involved in range finding is the speed of light. 3D clearance inspection for railway tunnels based on the TOF method has been used for many practical applications [6,7]. However, due to the intrinsic limitations of both the physical resolutions and sampling rates of TOF sensors, this method can hardly meet the expected demands of high-speed and high-accuracy dynamic inspection. The SV method is the approach to acquire 3D geometric information about an object’s surface according to two or more perspective images obtained by stereovision sensors. The operation of this kind of stereovision sensor mainly relies on the changes of the light reflection or radiation from the object surface. However, the smooth characteristics of railway tunnel surfaces make the extraction of the corresponding feature points from the perspective images results a challenge.

The LT method, by adopting a laser stripe modulated by the structured-light plane intersecting with the object, makes the extraction of feature points from the captured images facile and has the additional advantages of flexibility, fast on-site acquisition and high accuracy. Thus, it is quite suitable for quickly measuring objects’ surfaces. Recently, the strengths of vision sensing and computer vision have led to the development of the LT method for various surface detection applications. Examples include circuit board inspection [8], railway track profile inspection [9], seamless steel pipe straightness inspection [10,11], *etc*. Using the LT method to detect railway tunnel 3D clearance with a complete field of view (FOV), a multi-camera and structured-light vision system (MSVS) should be employed to capture the images of railway tunnel surfaces from different orientations, because the FOV of a single vision sensor is often too limited. When using the LT method to collect the 3D metric information of railway tunnels, other new problems arise, such as the lack of a common FOV and a wide distribution of the different cameras. These problems make the global calibration of MSVS a difficult task.

The vision principle of the LT method and global calibration for a multi-camera system has previously been presented in [5,12]. However, a detailed analysis of the imaging model of MSVS in railway tunnel 3D clearance dynamic inspection applications has not yet been performed, and the global calibration algorithm needs to be further explained in detail. Moreover, MSVSs for railway tunnel 3D clearance dynamic measurement are mounted on a dedicated vehicle. When the onboard vision sensing system begins to work, the vision sensors suffer from the multiple degrees of freedom vibrations caused by the running vehicle. In order to reduce the impact of vehicle vibrations on the measurement results, a compensation method to improve measurement accuracy should be employed and added to the software. In this paper, we focus on the two key issues in railway tunnel 3D clearance dynamic inspection: one is the global calibration of MSVS; the other is the compensation approach to reduce the measurement errors induced by vehicle vibrations.

The rest of this paper is organized as follows: Section 2 mainly describes the basic vision principle of MSVS for railway tunnel 3D clearance dynamic inspection and the MSVS parameters that need to be calibrated. Section 3 introduces the calibration approach in detail. The vehicle vibration compensation approach to improve the measurement accuracy is detailed in Section 4. Section 5 provides the experimental results performed by the proposed method. The conclusions are presented in Section 6.

## 2. Measurement Principle and Calibration Parameters

### 2.1. Measurement Principle

**Figure 1:**
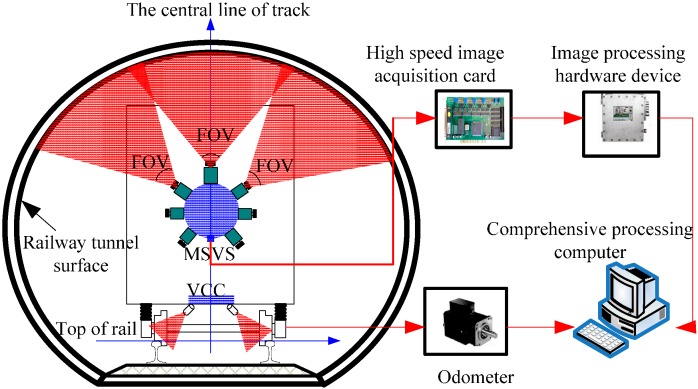
The vision measurement principle. MSVS, multi-camera and structured-light vision system; VCC, vibration compensation component.

The vision principle of railway tunnel 3D clearance dynamic inspection based on the LT method is illustrated in Figure 1. The measurement system includes MSVS, a high-speed image acquisition block, an odometer, a vibration compensation component (VCC) and an image processing computer:

(1) MSVS: including multiple cameras and structured-light projectors. The FOV of the cameras and projectors are overlapping in the measurement range.
(2) High-speed image acquisition block: used to collect images and send them to the computer.
(3) Odometer: including an optical-electrical encoder on the train axle transforming the turning angle of the axle to pulse signals and a signal controller calculating mileage from the number of pulses.
(4) VCC: including two line structured-light vision sensors installed at the bottom of the vehicle body to monitor the feature point of the railhead.

As shown in Figure 1, MSVSs are installed at the frontal-side of the vehicle body. The structured-light projectors emit laser planes from different orientations. The laser planes intersect with the surface of the railway tunnel and form laser stripes in the same cross-section with full FOV. When the train is running, the optical-electrical encoder emits pulse signals and sends them to the signal controller to calculate the mileage. Simultaneously, it generates trigger signals to control the different cameras of the MSVS working synchronously. The laser stripe images captured by the cameras are sent to the computer through the high-speed image acquisition block. Since the laser stripes are modulated by the depth of the tunnel surface, the image processing software can reconstruct the 3D metric information of the railway tunnel based on the optical triangulation principle.

For the railway tunnel 3D clearance dynamic inspection, one of the key issues is to extract the metric information of the railway tunnel from multiple 2D distorted stripe images. The high-accuracy global calibration of MSVS is the first step. The so-called global calibration of MSVS is the process of determining the mapping relationship between the 3D world coordinate frame and the 2D image coordinate frame based on the optical imaging model.

### 2.2. Calibration Approach

Due to the large range and complete FOV of MSVSs, traditional approaches [13,14,15,16,17,18,19,20,21,22,23] can hardly realize the global calibration of MSVSs. However, the intrinsic parameters of MSVSs are only determined by the sensors and lenses themselves, independent of their placement orientations and positions. According to this property, we can adopt a 2D chessboard as a calibration target to extract the intrinsic parameters of each camera off-line in advance. After the intrinsic parameters of each vision sensor have been obtained and the MSVS has been assembled, we then use a 1D target, which has the advantages of high accuracy, simple structure and easy manufacturing, to extract the extrinsic parameters of the MSVS on-line. Through the combination of a 2D planar target and a 1D target, we can ultimately realize the global calibration of the MSVS. In this paper, the proposed approach mainly contains the following four steps:

(1) For each camera, the perspective projective matrix and lens distortion coefficients are calibrated off-line by the 2D planar target.
(2) After, the MSVS is assembled, placing the 1D target to cover the FOV of two neighboring cameras and then computing the extrinsic parameters of each neighboring cameras on-line, including the rotation matrix and translation vector, according to the collinear property and known distances of feature points on the 1D target [24,25,26,27]. Then, an arbitrary camera coordinate frame is selected as the global coordinate frame. By utilizing the extrinsic parameters of each neighboring camera, we can transform the measurement results of the other cameras from their local coordinate frame to the global coordinate frame.
(3) Using the same computation method in Step 2 and through at least three non-collinear feature points on the structured-light plane, the equation of the structured-light plane can also be determined.
(4) With the help of the intrinsic parameters of each camera, the extrinsic parameters of the neighboring cameras and the structured-light plane equation, the global measurement model of the MSVS can ultimately be obtained.

## 3. The Global Calibration of the Vision System

This section briefly introduces the basic notations in the intrinsic parameter calibration process of each camera off-line by using a 2D planar target. Then, the extrinsic parameter calibration for the MSVS are detailed, starting with the rotation matrix and translation vector acquisition between the neighboring cameras, followed by the structured-light plane determination and ending with the global optimization for the vision system.

### 3.1. Basic Notations

**Figure 2:**
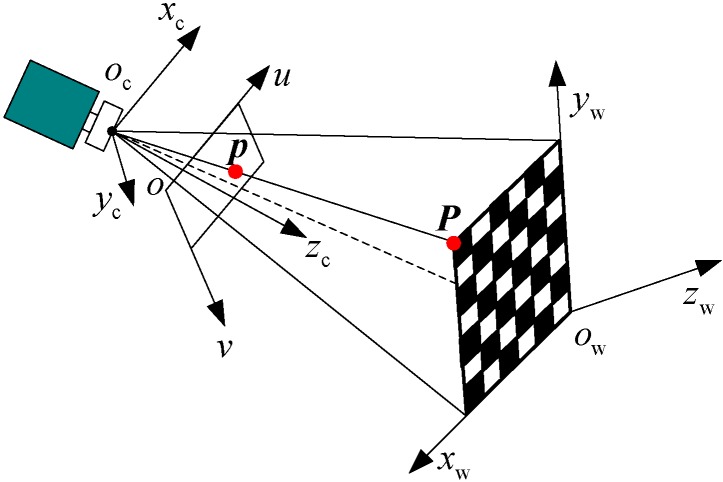
The intrinsic parameters calibration principle.

Without loss of generality, a usual pinhole camera model is used. As shown in Figure 2, a 2D image point is denoted by $p={\left(\begin{array}{cc} u & v \end{array}\right)}^{T}$ and a 3D world coordinates point by $P={\left(\begin{array}{ccc} x & y & z \end{array}\right)}^{T}$.

The corresponding homogeneous coordinates are written by $\tilde{p}={\left(\begin{array}{ccc} u & v & 1 \end{array}\right)}^{T}$ and $\tilde{P}={\left(\begin{array}{cccc} x & y & z & 1 \end{array}\right)}^{T}$. Based on the pinhole model, the mapping of a 3D world coordinates point to a 2D image point is described by:

(1) $$s\tilde{p}=A(\begin{array}{cc} R & t \end{array})\tilde{P},\;A=\left[\begin{array}{lll} \alpha & \gamma & u_{0}\\ 0 & \beta & v_{0}\\ 0 & 0 & 1 \end{array}\right]$$

where *s* is an arbitrary scale factor that is not equal to zero. ***A*** is called the intrinsic matrix, which contains five parameters: α and β are the scale factors in the image axes u and v, $(u_{0},v_{0}\text{)}$ is the principle point and γ is the skew of the two image axes, which in practice is almost always set to zero. $\left(\begin{array}{cc} R & t \end{array}\right)$, called the extrinsic matrix, is composed of a rotation matrix and a translation vector from the world coordinate frame to camera coordinates frame.

If the world coordinates are established on a plane (z-axis is the perpendicular), then the point on the plane is $\tilde{P}={\left(\begin{array}{cccc} x & y & 0 & 1 \end{array}\right)}^{T}$. Let us redefine $\tilde{P}$ as $\tilde{P}={\left(\begin{array}{ccc} x & y & 1 \end{array}\right)}^{T}$ and denote the *i*-th column of the rotation matrix R by $r_{i}$. From Equation (1), we have:

(2) $$s\tilde{p}=A(\begin{array}{ccc} r_{1} & r_{2} & t \end{array})\tilde{P}$$

According to the projective geometry, this plane to plane mapping can also be expressed by a projective transform:

(3) $$s\tilde{p}=H\tilde{P}$$

where H is a 3 × 3 homography matrix defined up to a scale factor. Let us denote the *i*-th column of H by $h_{i}$. From Equations (2) and (3), we have:

(4) $$\lambda (\begin{array}{ccc} h_{1} & h_{2} & h_{3} \end{array})=A(\begin{array}{ccc} r_{1} & r_{2} & t \end{array})$$

If A and H are known, then the extrinsic matrix $\left[\begin{array}{cc} R & t \end{array}\right]$ is readily computed. From Equation (4), we have:

(5) $$\begin{array}{l} r_{1}=\lambda A^{-1}h_{1},\;r_{2}=\lambda A^{-1}h_{2},\;r_{3}=r_{1}\times r_{2},\;t=\lambda A^{-1}h_{3}\\ \lambda =1/\left\|A^{-1}h_{1}\right\|=1/\left\|A^{-1}h_{2}\right\| \end{array}$$

### 3.2. Extrinsic Parameters Calibration of Neighboring Cameras

**Figure 3:**
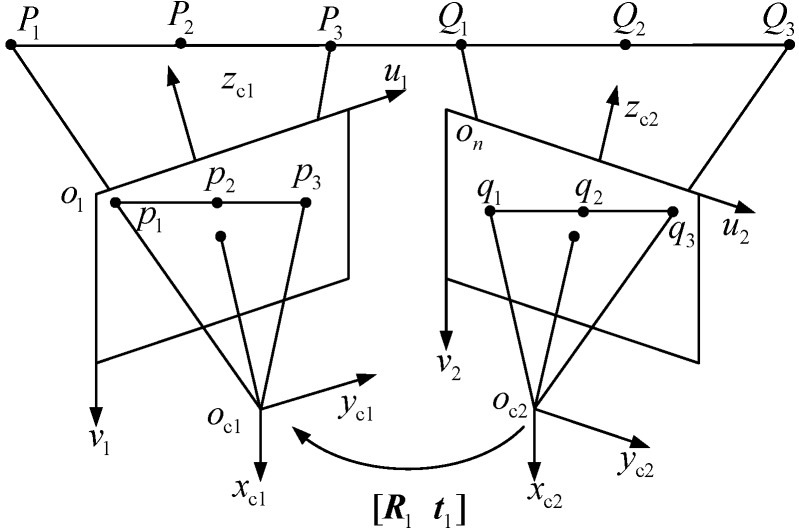
The extrinsic parameter calibration for neighboring cameras.

The extrinsic parameter $\left[\begin{array}{cc} R_{1} & t_{1} \end{array}\right]$ calibration principle of neighboring cameras using a 1D target is illustrated in Figure 3. The 1D target should have at least six collinear feature points guaranteeing that there are at least three feature points in the FOV of each vision sensor. The distance between different feature points was known previously.

As shown in Figure 3, the feature points in the camera coordinate frame $o_{c1}x_{c1}y_{c1}z_{c1}$ and $o_{c2}x_{c2}y_{c2}z_{c2}$ are denoted as $P_{j}$ and $Q_{j}$, respectively. Their image coordinates are denoted as $p_{j}$ and $q_{j}$. In addition, let ${\tilde{P}}_{j}$, ${\tilde{Q}}_{j}$, ${\tilde{p}}_{j}$ and ${\tilde{q}}_{j}$ denote the augment vector by adding one as the last element of $P_{j}$, $Q_{j}$, $p_{j}$ and $q_{j}$.

Assuming that the infinite point coordinate of the 1D target in camera coordinate frame $o_{c1}x_{c1}y_{c1}z_{c1}$ to be denoted as $P_{\infty }$, according to the cross ratio definition [28,29], the cross ratio of feature points $P_{1}$, $P_{2}$, $P_{3}$ and $P_{\infty }$ can be computed by Equation (6):

(6) $$\text{CR}(P_{1},P_{2},P_{3},P_{\infty })=\frac{\left||P_{1}P_{3}|\right|}{\left||P_{2}P_{3}|\right|}:\frac{\left||P_{1}P_{\infty }|\right|}{\left||P_{2}P_{\infty }|\right|}\approx \frac{\left||P_{1}P_{3}|\right|}{\left||P_{2}P_{3}|\right|},\text{ where}\frac{\left||{\text{P}}_{1}P_{\infty }|\right|}{\left||P_{2}P_{\infty }|\right|}\approx 1$$

The projective point of $P_{\infty }$ in the image coordinate frame is called the image vanishing point and is denoted as $p_{\infty }$. According to the invariance of the cross ratio by the perspective projection rule, we have:

(7) $$\text{CR}(p_{1},p_{2},p_{3},p_{\infty })=\text{CR}(P_{1},P_{2},P_{3},P_{\infty })\approx \frac{\left||P_{1}P_{3}|\right|}{\left||P_{2}P_{3}|\right|}$$

where $p_{1}$, $p_{2}$ and $p_{3}$ denote the corresponding image point of 3D point $P_{1},P_{2},P_{3}$. When the 1D target images are captured by each camera, the image coordinates of feature point $p_{1},p_{2}$ and $p_{3}$ can be accurately determined by the sub-pixel extracting algorithm [30,31]. Because the distance of $\left||P_{1}P_{3}|\right|$ and $\left||P_{2}P_{3}|\right|$ are given, the coordinate of image vanishing point $p_{\infty }$ can be computed by Equation (7).

Assuming that the 1D target can be freely moved, when the 1D target is at the *i*-th position, the 1D target unit directional vector, in the *k*-th camera coordinate frame, is denoted as $n_{ki}$. The image vanishing point is denoted as $p_{ki}$. Through Equation (7), we can get the image coordinates of vanishing point $p_{ki}$ [32,33,34].

(8) $$n_{ki}=\frac{A_{k}^{-1}p_{ki}}{\left\|A_{k}^{-1}p_{ki}\right\|},\text{ where }k=1,2$$

When the 1D target is placed at the *i*-th position, the unit directional vectors determined by the 1D target in the camera coordinates $o_{c1}x_{c1}y_{c1}z_{c1}$, $o_{c2}x_{c2}y_{c2}z_{c2}$ are denoted as $n_{1i}$ and $n_{2i}$. The unit directional vector $n_{1i}$ and $n_{2i}$ can be easily computed through Equation (8) by the combination of image vanishing points $p_{1i}$, $p_{2i}$ and intrinsic parameters $A_{1}$ and $A_{2}$. Then, $n_{1i}$ and $n_{2i}$ can be related by the rotation matrix $R_{1}$, *i.e*., $n_{2i}=R_{1}n_{1i}$. To extract the rotation matrix $R_{1}$, the 1D target should be moved at least two times. Thus, there exists Equation (9):

(9) $$\left\{ \begin{array}{l} n_{21}=R_{1}n_{11}\\ n_{22}=R_{1}n_{12}\\ n_{21}\times n_{22}=R_{1}(n_{11}\times n_{12}) \end{array}\right.$$

If $\text{rank}[n_{21}\;n_{22}\;n_{21}\times n_{22}]=3$, we can get the rotation matrix $R_{1}$ through Equation (10):

(10) $$R_{1}=[\begin{array}{ccc} n_{21} & n_{22} & n_{21}\times n_{22} \end{array}]{\left[\begin{array}{ccc} n_{11} & n_{12} & n_{11}\times n_{12} \end{array}\right]}^{-1}$$

When the 1D target is moved to the *i*-th position, the feature point $P_{j}$ is denoted as $P_{ji}$. The image coordinates of $P_{ji}$ is denoted as $p_{ji}$, and the augment vector is denoted as ${\tilde{p}}_{ji}$. With the help of the intrinsic parameters matrix $A_{1}$, we can get the following relation between $P_{ji}$ and ${\tilde{p}}_{ji}$ as Equation (11):

(11) $$\left\{ \begin{array}{l} x_{ji}={\left(A_{1}^{-1}{\tilde{p}}_{ji}\right)}_{1}z_{ji}\\ y_{ji}={\left(A_{1}^{-1}{\tilde{p}}_{ji}\right)}_{2}z_{ji} \end{array}\right.$$

When the 1D target is at the *i*-th position, the coordinates of feature points $P_{1i}$, $P_{2i}$ are denoted as $\left(x_{1i},y_{1i},z_{1i}\right)$ and $\left(x_{2i},y_{2i},z_{2i}\right)$ and can be expressed as Equations (12) and (13), respectively:

(12) $$\left\{ \begin{array}{l} x_{1i}={\left(A_{1}^{-1}{\tilde{p}}_{1i}\right)}_{1}z_{1i}\\ y_{1i}={\left(A_{1}^{-1}{\tilde{p}}_{1i}\right)}_{2}z_{1i} \end{array}\right.$$

(13) $$\left\{ \begin{array}{l} x_{2i}={\left(A_{1}^{-1}{\tilde{p}}_{2i}\right)}_{1}z_{2i}\\ y_{2i}={\left(A_{1}^{-1}{\tilde{p}}_{2i}\right)}_{2}z_{2i} \end{array}\right.$$

The unit directional vector $n_{1i}$ in the camera coordinate frame $o_{c1}x_{c1}y_{c1}z_{c1}$ is specified as (dx,dy,dz). Thus, $P_{1}P_{2}$ can be written as:

(14) $$\frac{x_{1i}-x_{2i}}{dx}=\frac{y_{1i}-y_{2i}}{dy}=\frac{z_{1i}-z_{2i}}{dz}$$

Since the distance of $P_{1}P_{2}$ is known, we can obtain Equation (15).

(15) $$\left[\begin{array}{ll} {\left(A_{1}^{-1}{\tilde{p}}_{1i}\right)}_{1} & -{\left(A_{1}^{-1}{\tilde{p}}_{2i}\right)}_{1}\\ {\left(A_{1}^{-1}{\tilde{p}}_{1i}\right)}_{2} & -{\left(A_{1}^{-1}{\tilde{p}}_{2i}\right)}_{2}\\ 1 & -1 \end{array}\right]\left[\begin{array}{l} z_{1i}\\ z_{2i} \end{array}\right]=L_{12}\left[\begin{array}{l} dx\\ dy\\ dz \end{array}\right]$$

From Equation (15), we can directly solve the unknown parameters $z_{1i}$ and $z_{2i}$. Then, substituting $z_{1i}$ and $z_{2i}$ into Equations (12) and (13), the coordinates $\left(x_{1i},y_{1i},z_{1i}\right)$ and $\left(x_{2i},y_{2i},z_{2i}\right)$ can be accurately computed.

Similarly, we can get the coordinates of $Q_{1i}$, $Q_{2i}$ and $Q_{3i}$ in the camera coordinate frame $o_{c2}x_{c2}y_{c2}z_{c2}$. Let us denote $Q_{ji}$ in the camera coordinate frame $o_{c1}x_{c1}y_{c1}z_{c1}$ as ${\hat{Q}}_{ji}$. Thus, there exists the formula ${\hat{Q}}_{ji}=R_{1}Q_{ji}+t_{1}$ that transforms feature point $Q_{ji}$ from camera coordinate frame $o_{c2}x_{c2}y_{c2}z_{c2}$ to $o_{c1}x_{c1}y_{c1}z_{c1}$. Therefore, the distance between $P_{ji}$ and ${\hat{Q}}_{ji}$ with reference to $o_{c1}x_{c1}y_{c1}z_{c1}$ can be computed by Equation (16):

(16) $$||P_{ji}-{\hat{Q}}_{ji}||\;=\;||P_{ji}-R_{1}Q_{ji}-t_{1}||\;=\;L_{P_{j}Q_{j}}$$

In Equation (16), $L_{P_{j}Q_{j}}$ is the known distance between the feature points $P_{j}$ and $Q_{j}$ on the 1D target, and $t_{1}$ is the only unknown variable. Solving Equation (16), the translation vector $t_{1}$ can be accurately extracted.

### 3.3. Structured-Light Plane Equation Calibration

**Figure 4:**
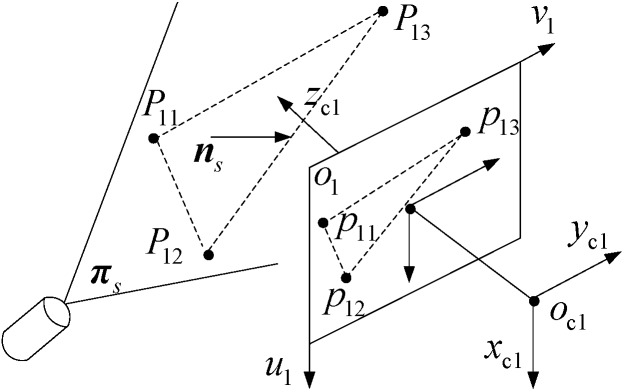
The calibration principle of the structured-light plane.

The principle of structured-light plane calibration in MSVS is illustrated as Figure 4. Let the 1D target, which contains at least three collinear points with known distance, be freely moved on the structured-light plane more than two times. The camera captures the laser stripe images generated by the line structured-light plane intersecting with the 1D target. Then, using the image sub-pixel processing algorithm extracts the center of the laser stripes with high-accuracy. By notation, we still use $P_{ji}$ to denote the feature points on the structured-light plane. The coordinate of $P_{ji}$ with reference to $o_{c1}x_{c1}y_{c1}z_{c1}$ can be obtained by the method provided in Section 3.2.

Assume that there exist three non-collinear feature points denoted as $P_{11}$, $P_{12}$ and $P_{13}$ on the structured-light plane, and the normal unit vector of the structured-light plane is denoted as $\pi_{s}$. Thus, we can compute $n_{s}$ as follow: $n_{s}=\left(P_{11}P_{12}\times P_{11}P_{13}\right)/\left||P_{11}P_{12}\times P_{11}P_{13}|\right|=[\begin{array}{ccc} x_{s} & y_{s} & z_{s} \end{array}]$. After $n_{s}$ is obtained, the structured-light plane equation can be uniquely determined by Equation (17):

(17) $$n_{s}(P_{c1}-P_{ji})=0$$

where $P_{c1}$ is an arbitrary point on the laser plane and $P_{ji}$ is a feature point whose coordinates are known on the structured-light plane. If $P_{c1}$ is denoted as ${\left[x_{c1}\;y_{c1}\;z_{c1}\right]}^{T}$, then Equation (17) can be rewritten as $ax_{c1}+by_{c1}+cz_{c1}+d=0$. If the number of feature points on the laser plane is more than three, the least squares method in [35,36] should be employed to get the optimization solution of $n_{s}$.

### 3.4. Global Optimization

The MSVS for railway tunnel 3D clearance dynamic inspection consists of multiple vision sensors. After implementing the aforementioned calibration procedures, we have obtained the intrinsic parameters of each camera, the extrinsic parameters of the neighboring camera and the laser plane structured parameters. The global measurement model of the MSVS is still not established. The task of this section is to unify the local coordinate frames of each camera to be under one global coordinate frame and to achieve the establishment of the MSVS global measurement model for railway tunnel 3D clearance dynamic inspection.

**Figure 5:**
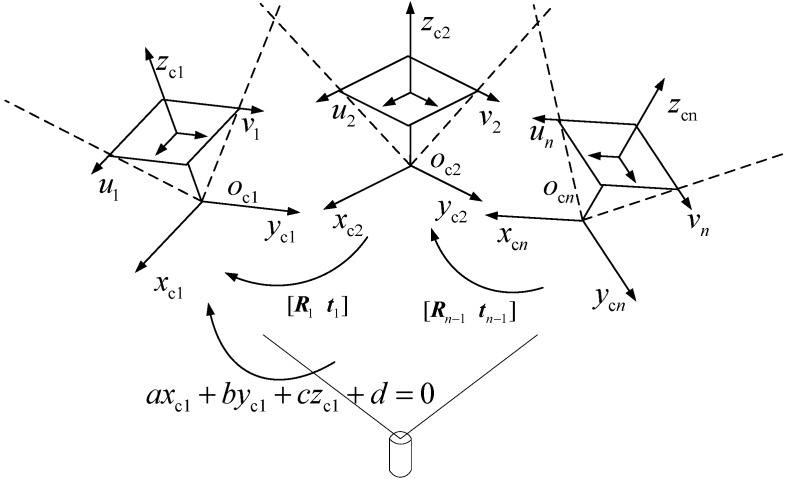
The global calibration principle of the MSVS.

The global optimization principle of the MSVS is shown in Figure 5. Selecting $o_{c1}x_{c1}y_{c1}z_{c1}$ as the global coordinate frame, the local coordinate frames of other n−1 cameras should be transformed into $o_{c1}x_{c1}y_{c1}z_{c1}$. The coordinate frame of the *n*-th camera is denoted as $o_{cn}x_{cn}y_{cn}z_{cn}$ and the corresponding image coordinate frame as $o_{n}x_{un}y_{un}$. The extrinsic parameters of the neighboring cameras are denoted as $\left[\begin{array}{cc} R_{n-1} & t_{n-1} \end{array}\right]$. The extrinsic parameters between the *n*-th camera coordinate frame and the global coordinate frame are denoted as $\left[\begin{array}{cc} {\hat{R}}_{n-1} & {\hat{t}}_{n-1} \end{array}\right]$.

In Section 3.2, the extrinsic parameters $\left[\begin{array}{cc} R_{1} & t_{1} \end{array}\right]$ of the neighboring cameras have been obtained. The same approach can also be put into the extraction of $\left[\begin{array}{cc} R_{n-1} & t_{n-1} \end{array}\right]$. Thus, the *n*-th camera local coordinate frame $o_{cn}x_{cn}y_{cn}z_{cn}$ can be converted into the global coordinate frame $o_{c1}x_{c1}y_{c1}z_{c1}$, through thefollowing transformations:

(18) $${\left[\begin{array}{ccc} x_{cn} & y_{cn} & z_{cn} \end{array}\right]}^{T}=[\begin{array}{cc} {\hat{R}}_{n-1} & {\hat{t}}_{n-1} \end{array}]{\left[\begin{array}{cccc} x_{c1} & y_{c1} & z_{c1} & 1 \end{array}\right]}^{T}=\left[\begin{array}{cc} \overset{n-1}{\underset{i=0}{\Pi }}R_{i-1} & \sum_{i=0}^{n-1}t_{i-1} \end{array}\right]{\left[\begin{array}{cccc} x_{c1} & y_{c1} & z_{c1} & 1 \end{array}\right]}^{T}$$

If we add the perspective imaging model into Equation (18), we can get Equation (19):

(19) $$\lambda_{n}{\left[\begin{array}{ccc} x_{un} & y_{un} & 1 \end{array}\right]}^{T}=A_{n-1}\left[\begin{array}{cc} \overset{n-1}{\underset{i=0}{\Pi }}R_{i-1} & \sum_{i=0}^{n-1}t_{i-1} \end{array}\right]{\left[\begin{array}{cccc} x_{c1} & y_{c1} & z_{c1} & 1 \end{array}\right]}^{T}$$

where $\lambda_{n}$ and $A_{n-1}$ are the scale factor and camera perspective matrix of the *n*-th camera. From Equation (19), it can be seen that if arbitrary image coordinates $\left(x_{un},y_{un}\right)$ from the MSVS are given, its 3D coordinates in the global coordinate frame $o_{c1}x_{c1}y_{c1}z_{c1}$ can be accurately computed.

## 4. Vehicle Vibration Compensation

Due to the fact that the MSVS is mounted on a running vehicle, the onboard vision sensors should suffer from multiple degrees of freedom vibrations brought about by the running vehicle. Any slight vibration can result in substantial measurement errors. If the dynamic inspection is implemented in curved railway lines, the measurement performance should be degraded much more severely. In order to reduce the measurement errors caused by the vehicle vibrations, an appropriate compensation method for vehicle vibrations should be adopted and added to the software to improve accuracy.

**Figure 6:**
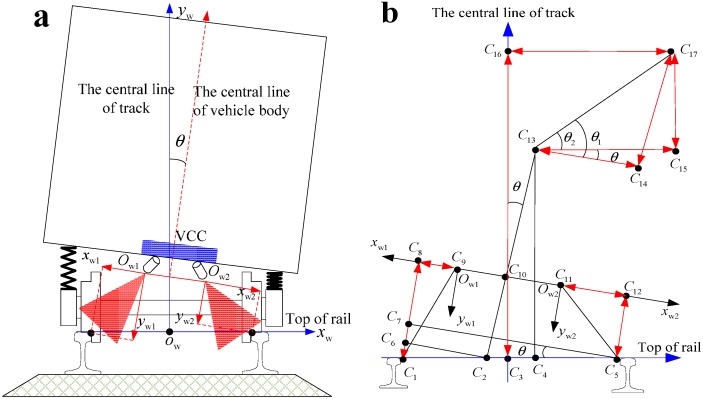
(**a**) The vehicle vibrations compensation principle; (**b**) the detailed computation sketch diagram.

The compensation principle of vehicle vibrations is shown in Figure 6. It can be seen that the VCC consists of two line structured-light vision sensors installed at the bottom of the vehicle body.

The structured-light vision sensors project line structured-light on the rails, ensuring that light sheds on the rail waist and rail head. The laser strip images are acquired by the high-speed image acquisition block and stored in the computer memory unit. The inspection software processes the laser stripe images and extracts rail feature points on-line. The rail feature point extraction algorithm can be found in [13,14]. The varied results of the rails’ features draws attention to the vision sensors’ local calibration coordinate frame, which can be obtained according to the laser triangulation principle.

When the dedicated inspection vehicle is running, the rolling vibration, pitching vibration and heading vibration are created simultaneously from the vehicle damping springs. Since these three types of vibrations are orthogonal to one another and the structured-light planes emitted by the projectors are perpendicular to the vehicle running direction, only the rolling vibration has a significant impact on the railway tunnel 3D clearance dynamic inspection measurement results. In this paper, the rolling angle is denoted as θ in Figure 6a. In the rest of this paper, we only consider the influence of the rolling vibration on the measurement results.

The compensation principle of vehicle vibrations is illustrated in Figure 6b. The definition of different notations is listed in Table 1. The parameters in Table 1 can be divided into three categories. The first category indicates the ranging results that can be directly obtained by the vision sensors of the VCC and MSVS, including $C_{1}C_{8}$, $C_{8}C_{9}$, $C_{5}C_{12}$, $C_{11}C_{12}$ and $C_{14}C_{17}$, $C_{13}C_{14}$. The second is the constants that are determined by the VCC and MSVS on-site installation, including $C_{9}C_{11}$ and $C_{10}C_{13}$. The third is the unknown parameters that need to be determined, including rolling angle θ, $C_{16}C_{17}$ and $C_{16}C_{13}$. If we do not add the vehicle vibration compensation algorithm to the measurement results, the detected values of the feature point $C_{17}$ are $\left(C_{13}C_{14},C_{14}C_{17}+C_{2}C_{13}\right)$. In fact, the real measurement coordinates in reference to the track central coordinate frame are $\left(C_{16}C_{17},C_{3}C_{16}\right)$. Vehicle vibration compensation is the goal of acquiring the coordinates of the feature points, *i.e*., $\left(C_{16}C_{17},C_{3}C_{16}\right)$, based on the parameters from the first and second category.

**Table 1:** The definitions of different notations.

Notation	Parameters
$C_{1}$	The feature point of the left rail
$C_{2}$	The intersection point of the vehicle central line and the rail top surface
$C_{3}$	The middle point of $C_{1}C_{5}$
$C_{4}$	The vertical intersection point of $C_{1}C_{5}$ and the line through $C_{13}$
$C_{5}$	The feature point of the right rail
$C_{6}$	The vertical intersection point of $C_{1}C_{8}$ and the line through $C_{2}$
$C_{7}$	The vertical intersection point of $C_{1}C_{8}$ and the line through $C_{5}$
$C_{8}$	The vertical intersection point of $C_{9}C_{11}$ and the line through $C_{1}$
$C_{9}$	The left calibration center of the VCC
$C_{10}$	The middle point of $C_{9}C_{11}$
$C_{11}$	The right calibration center of the VCC
$C_{12}$	The vertical intersection point of $C_{9}C_{11}$ and the line through $C_{5}$
$C_{13}$	The calibration center of the MSVS
$C_{17}$	An arbitrary feature point on the surface of the railway tunnel
$C_{3}C_{16}$	The central line of the track
$C_{10}C_{13}$	The central line of the vehicle body
$C_{1}C_{8}$	The vertical ranging result of VCC for the left rail
$C_{8}C_{9}$	The horizontal ranging result of VCC for the left rail
$C_{5}C_{12}$	The vertical ranging result of VCC for the right rail
$C_{11}C_{12}$	The horizontal ranging result of VCC for the right rail
$C_{14}C_{17}$	The vertical ranging result of the MSVS for the railway tunnel
$C_{13}C_{14}$	The horizontal ranging result of the MSVS for the railway tunnel
θ	The vehicle rolling vibration angle

In Section 3, the calibration approach for the intrinsic and extrinsic parameters of the MSVS has been detailed. Similarly, this approach is also suitable for the calibration of the VCC. Therefore, we can establish the imaging model for the two compensation vision sensors through Equations (20) and (21):

(20) $$\left\{ \begin{array}{l} x_{w1}=\varphi_{x1}(x,y)\\ y_{w1}=\varphi_{y1}(x,y) \end{array}\right.$$

(21) $$\left\{ \begin{array}{l} x_{w2}=\varphi_{x2}(x,y)\\ y_{w2}=\varphi_{y2}(x,y) \end{array}\right.$$

where (x,y) denotes the image coordinates of the rail feature points $C_{1}$ and $C_{5}$. The coordinates of $C_{1}$ and $C_{5}$ in their local measurement coordinate frame are denoted as $\left(x_{w1},y_{w1}\right)$ and $\left(x_{w2},y_{w2}\right)$, which are illustrated in Figure 6b.

Using the imaging model provide by Equations (20) and (21), we can carry out the vehicle vibration compensation algorithm as the following four steps:

(1) Computing the rolling vibration angle and the auxiliary angles:
  $$\theta =\text{atan}(\frac{C_{1}C_{8}-C_{5}C_{12}}{C_{8}C_{9}+C_{9}C_{11}+C_{11}C_{12}});\;\theta_{1}=\text{atan}(\frac{C_{14}C_{17}}{C_{13}C_{14}});\theta_{2}=\theta_{1}-\theta .$$
(2) Decomposing $C_{13}C_{17}$ orthogonally in the track central coordinate frame:
  $$C_{15}C_{17}=\sqrt{{\left(C_{14}C_{17}\right)}^{2}+{\left(C_{13}C_{14}\right)}^{2}}\cdot \sin\;\theta_{2};\;C_{13}C_{15}=\sqrt{{\left(C_{14}C_{17}\right)}^{2}+{\left(C_{13}C_{14}\right)}^{2}}\cdot \cos\;\theta_{2}.$$
(3) Computing the ranging variables $C_{4}C_{13}$ and $C_{3}C_{4}$:
  $$C_{4}C_{13}=[C_{10}C_{13}+C_{1}C_{8}-(C_{8}C_{9}+0.5C_{9}C_{11})\cdot \text{tan }\theta ]\cdot \text{cos }\theta ;$$
  $$C_{3}C_{4}=[C_{10}C_{13}+C_{1}C_{8}-(C_{8}C_{9}+0.5\cdot C_{9}C_{11})\cdot \text{tan }\theta ]\cdot \text{sin }\theta +\frac{\left(C_{8}C_{9}+0.5\cdot C_{9}C_{11}\right)}{\text{cos }\theta }-\frac{C_{1}C_{5}}{2}.$$
(4) Computing the coordinates of an arbitrary feature point on the surface of the railway tunnel reference to the track central coordinate frame:
  $$C_{3}C_{16}=C_{15}C_{17}+C_{4}C_{13},\;C_{16}C_{17}=C_{13}C_{15}+C_{3}C_{4}.$$

After the four transformations, the real coordinate values of an arbitrary point on the surface of the railway tunnel are accurately obtained, and the measurement errors caused by the vehicle vibrations can be ultimately eliminated.

## 5. Experimental Section

In this section, the experiments consist of two parts: one is the calibration of the vision sensors from the MSVS; the other is the railway 3D clearance dynamic measurement. The details are presented in Section 5.1 and Section 5.2, respectively.

### 5.1. Calibration Experiments

In this section, a MICROVIEW camera Model MVC1000SAM_GE60 with 1280 × 1024 resolution, a KOWA 5-mm focal length lens Model LM5NCL and a Z_Laser Model ZM18 are selected to establish the vision system for railway tunnel 3D clearance dynamic inspection. The MSVS, consisting of seven CCD cameras and seven line structured-light projectors, and the VCC, consisting of two CCD cameras and two line structured-light projectors, are calibrated by the proposed approach.

**Figure 7:**
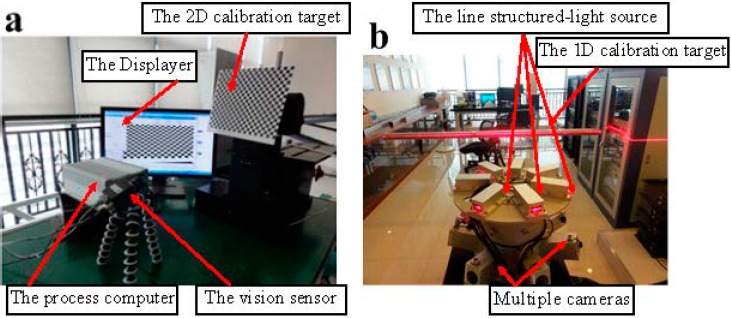
(**a**) The intrinsic parameter calibration using the 2D planar target off-line; (**b**) the extrinsic parameter calibration using the 1D target on-line.

Before implementing the MSVS assembly procedure, the intrinsic parameter calibration of each camera should be performed first. As shown in Figure 7a, a chessboard, which contains 25 × 20 squares with a distance between the near square corners of 10 mm, is applied in the off-line calibration of the camera intrinsic parameters. In the FOV of each camera, it is only required for the camera to observe the planar target pattern shown at different orientations. In this paper, each camera captures eight images of the planar pattern. Then, with the help of the Harris corner extraction [37] and Zhang’s calibration [18] algorithms, the intrinsic parameters of each camera can be accurately obtained. Due to paper space limitations, the planar pattern images applied in the intrinsic parameters calibration are not carried out, and we only present the acquisition results of the intrinsic parameters in Table 2, where α and β are the scale factors in the image axes u and v, $\left(u_{0},v_{0}\right)$ is the principle point and γ is the skew factor of the two image axes.

**Table 2:** The intrinsic parameter calibration results.

Parameters	Camera 1	Camera 2	Camera 3	Camera 4	Camera 5	Camera 6	Camera 7
α	2265.17	2268.45	2262.75	2267.32	2268.89	2260.77	2269.53
β	2268.23	2269.23	2266.91	2265.41	2265.42	2265.92	2267.48
γ	−0.81	1.08	−1.34	−0.88	1.07	−1.14	−0.56
$u_{0}$	639.24	645.32	640.25	639.45	637.46	637.55	643.55
$v_{0}$	518.05	516.41	513.25	511.90	514.12	513.51	516.02

In Table 2, we have found that the results are very consistent with each other. In order to further investigate the stability of the proposed calibration algorithm, we have presented the nominal values of this type of camera, where α = 2264.1, β = 2264.1, γ = 0, $u_{0}$ = 640 and $v_{0}$ = 512, as provided by the manufacturer. Comparing the nominal values with the calibration results of Table 2, it can be seen that the sample deviations for all parameters are quite small, which implies that the proposed algorithm is quite stable. The value of the skew parameters γ is not significant from 0, since the coefficients of variation, from −1.34–1.08, are quite large. Indeed, the maximum value γ = −1.34 with α = 2262.75 corresponds to 89.97 degrees, which is very close to 90 degrees, for the angle between the two image axes. We have also computed the aspect ratio α/β. It is very close to 1, *i.e*., the pixels are square.

After finishing the intrinsic parameter extraction of each vision sensor, the extrinsic parameters of the MSVS can be obtained according to the acquisition results of Table 2, and the next step is to calibrate the extrinsic parameters of the MSVS. The setup for the extrinsic parameter calibration is shown in Figure 7b. Without loss of generality, multiple cameras can be disassembled into several couples, and the neighboring cameras are calibrated individually.

**Figure 8:**
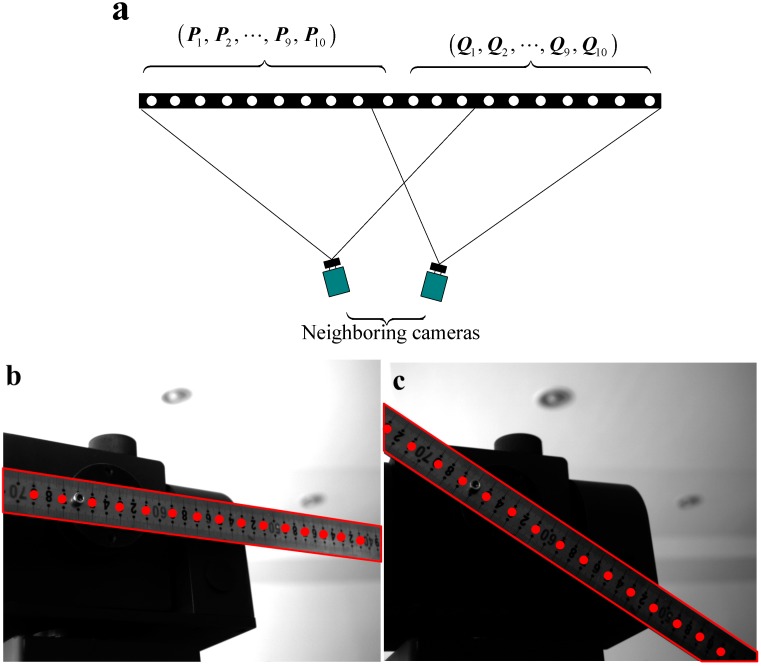
(**a**) The 1D target for the camera extrinsic parameter calibration; (**b**) the 1D target image for a one-camera calibration; (**c**) the 1D target image for a one-camera calibration.

As shown in Figure 8a, on the surface of the 1D target, there is a total of 20 feature points. In the calibration of each camera couple, the 1D target is fixed and crosses through the FOV of the neighboring cameras. Then, each camera captures part of the 1D target, and the same 1D target imaged by the neighboring cameras is used in the extrinsic parameters’ extraction. In Figure 8a, it can be seen that the left vision sensor captures feature Points 1–10, and the right vision sensor captures feature Points 11–20.

In order to guarantee the measurement accuracy of the vision system, all of the vision sensors collect the calibration images from multiple views. In the actual calibration experiment, the 1D target is firstly placed in front of each neighboring vision sensor 12 times with different states, and each vision sensor captures 12 images with the same 1D target at different orientations. Then, selecting the coordinate frame of Camera 1 as the global coordinate frame, the 1D target is also moved 12 times in the FOV of the global camera. At each pose, the structured-light plane intersects with the 1D target and formats a laser stripe on its surface, while the global camera captures a total of 12 images with laser stripes.

Due to the limitation space, we only show the images captured by one vision sensor when the 1D target is placed at two different poses. The obtained images are shown in Figure 8b and Figure 8c. By use of the total images and the proposed global calibration method of Section 3, the extrinsic parameters of each neighboring camera and the structured-light plane equation with reference to the global coordinate frame can be directly obtained. The obtained result is calculated as follows:

$$x_{c1}+0.012y_{c1}-0.036z_{c1}+256.45=0.$$

Finally, we can use the global optimization method provided by Section 3.4 to compute the camera extrinsic parameter matrix $\left[\begin{array}{cc} {\hat{R}}_{n-1} & {\hat{t}}_{n-1} \end{array}\right]$ and transform the coordinates of an arbitrary feature point from its local coordinate frame to the global coordinate frame. Then, by utilizing of the intrinsic parameters of each camera and the structured-light plane equation, the global measurement model of the MSVS for the railway tunnel 3D clearance dynamic inspection can ultimately be obtained.

### 5.2. Dynamic Inspection Experiments

**Figure 9:**
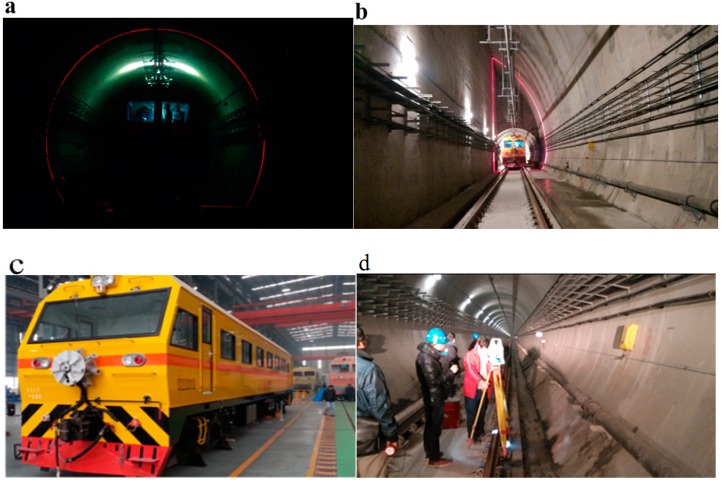
(**a**) The circle tunnel; (**b**) the half-circle tunnel; (**c**) the dedicated vehicle installed with the MSVS and VCC; (**d**) the manual static measurement.

In order to validate the effectiveness of the proposed approach in the MSVS calibration, experiments were conducted in the field on a metro line. The dedicated vehicle with the installed MSVS and VCC for railway tunnel 3D clearance dynamic inspection is shown in Figure 9. In the dynamic measurement, the inspection software collects each full cross-sectional profile of the railway tunnel with an interval of 250 mm at a speed of about 60 km/h. There mainly exist three clearance shapes of metro tunnels in China, e.g., circle tunnels, half-circle tunnels and rectangular tunnels.

The experiments are implemented in the region between People’s Square Station and Jiansheyi Road Station of Hangzhou Metro Line 2. In this region, the investigated tunnels include two different types, such as circle tunnels and rectangular tunnels.

**Figure 10:**
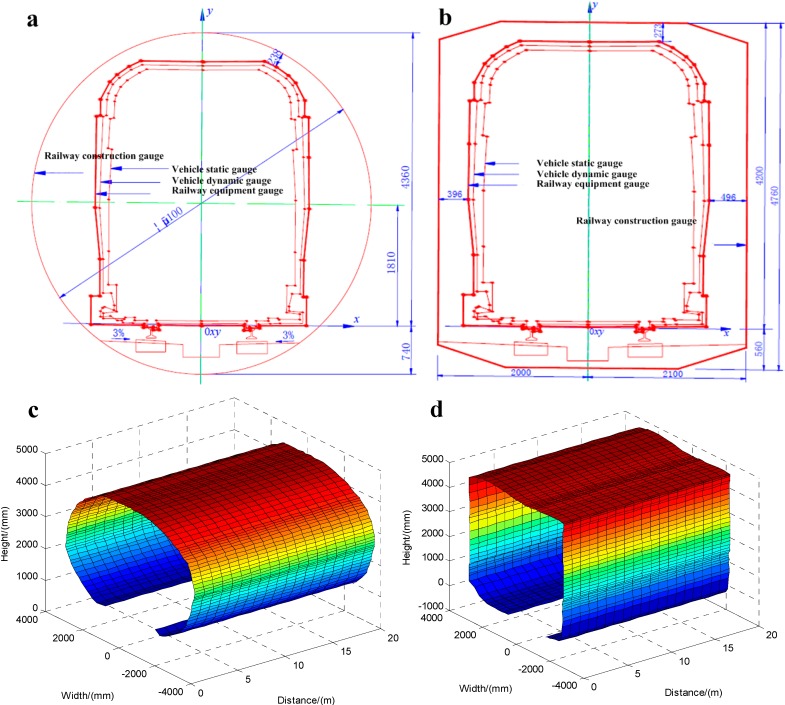
(**a**) The circle tunnel actual drawing; (**b**) the rectangle tunnel actual drawing; (**c**) the circle tunnel dynamic measurement results; (**d**) the rectangle tunnel dynamic measurement results.

Since the tunnel of this metro line is designed according to Chinese construction standards, the corresponding drawings showing the geometric dimensions of circle and rectangle tunnels can be separately carried out in Figure 10a,b according to [1]. The drawing of each type of railway tunnel contains vehicle static and dynamic geometric gauges, railway equipment and construction geometric gauges, which are all plotted and labeled individually.

In one complete cross-sectional profiling dataset of the railway tunnel, there is a total of 8960 feature points, which are collected by the vision sensors of the MSVS simultaneously. In the experiments for railway tunnel 3D clearance dynamic measurements, the continuous records consisting of circle and rectangle tunnels within a 20-m distance are acquired by the inspection software automatically and are shown in Figure 10c,d, respectively. Based on the results shown in Figure 10c,d, we can directly get the dimensional information of the railway tunnel’s 3D clearance. Furthermore, if we compared the measurement results of Figure 10c,d with the drawings of Figure 10a,b, a slight deformation on the surface of the railway tunnel can be found over time, which can eliminate potential risks, avoid incidents and also reduce maintenance costs.

From Figure 10c,d, it can be seen that the surfaces of the 3D images are smooth and without any foreign objects on them. After the comparison of the measurement results of Figure 10c,d and the design drawings of Figure 10a,b, we find that there is no obvious deformation on the surfaces, and the railway tunnels in these areas are in good order.

**Figure 11:**
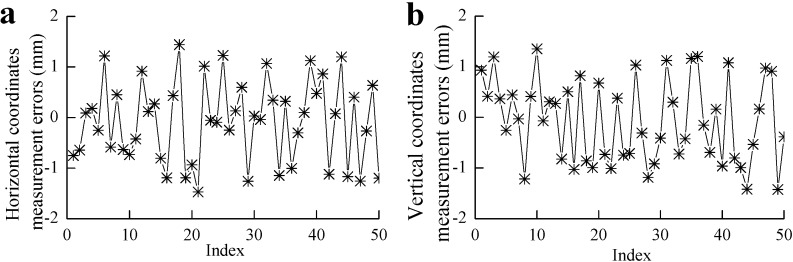
(**a**) The horizontal coordinate measurement errors; (**b**) the vertical coordinate measurement errors.

Furthermore, in order to determine the measurement accuracy of the vision system, 50 feature points, evenly distributed over 50 different cross-sections of railway tunnel along a 20 m section, are selected as testing feature points. Through Figure 10c, the coordinates of these feature points measured by the dynamic vision system are obtained. Then, with the help of a manually-operated theodolite, we can get the coordinates of these feature points under the static state with 0.5-mm accuracy. Selecting the manual measurement results as the basic data and comparing the dynamic measurement results with them, the dynamic measurement errors of these feature points in the horizontal and vertical directions can be easily obtained. The coordinate measurement errors of these feature points in the horizontal and vertical directions are calculated in Figure 11a,b, respectively.

According to the results shown in Figure 11a,b, it is clear that the measurement errors are not completely eliminated, although the vehicle vibration compensation algorithm is added to the measurement results. From Figure 11a,b, it is not difficult to find that both of the errors in the horizontal and vertical directions conform to a random distribution, and the centers of the random distributions are approximately zero. Since the mean values of the measurement results approach zero, we can conclude that the systemic errors of the dynamic measurement results are almost eliminated, and the current errors are mainly increased by the random errors, which may be brought about by the noises in the procedures of vision sensor calibration and dynamic measurements.

In order to quantitatively analyze the measurement errors of the vision system, the minimum measurement errors, the maximum measurement errors and the root mean square (RMS) measurement errors from the 50 sample feature point error results of Figure 11 in the horizontal and vertical directions are computed. Let $e_{\min}$, $e_{\max}$ and $e_{\text{RMS}}$ denote the minimum error, maximum error and RMS error, respectively. The statistical results of the measurement errors based on Figure 11b are detailed in Table 3.

From Table 3, it can be seen that the maximum measurement error is −1.47 mm. In the railway tunnel field dynamic measurement, the required accuracy is 5 mm. The results of Figure 11a,b show that the dynamic measurement errors are within the allowed range and also demonstrate that the vision system can fully satisfy railway tunnel 3D clearance field measurement.

**Table 3:** The measurement errors.

Notation	$e_{\min}$ (mm)	$e_{\max}$ (mm)	$e_{\mathrm{RMS}}$ (mm)
Horizontal measurement errors	0.12	−1.47	0.81
Vertical measurement errors	−0.10	1.43	0.82

## 6. Conclusions

In this paper, a global calibration method for an MSVS is presented. The use of a 2D planar target to calibrate the intrinsic parameters of each camera off-line and the 1D target to calibrate the extrinsic parameters of the neighboring cameras, as well as the equation for the on-line structured-light plane, respectively, is proposed. By integration of the intrinsic parameters to each camera, the extrinsic parameters of each neighboring camera and the structured-light plane equation, the global measurement model can be successfully established. The onboard vision system is mounted on a dedicated vehicle, and it is expected to suffer from multiple degrees of vibrations caused by the running vehicle. In order to overcome this problem, a vehicle motion deviation rectifying method is proposed. The minimum errors, maximum errors and RMS errors based on the railway tunnel 3D clearance dynamic inspection results are calculated, which demonstrate the effectiveness of the proposed vision system.
